# Evaluation of crestal sinus floor elevation in cases exhibiting an oblique sinus floor with a residual bone height of 4.0–7.0 mm using Densah burs with simultaneous implant placement: a prospective clinical study

**DOI:** 10.1186/s40729-023-00510-1

**Published:** 2023-11-02

**Authors:** Mahmoud Shalash, Mohamed Mounir, Tala Elbanna

**Affiliations:** 1 Surgery and Oral Medicine Department, National Research Centre, Cairo, Egypt; 2 Oral and Maxillofacial Surgery Department, Faculty of Dentistry, Cairo University, Cairo, Egypt; 3 Ministry of Health, Cairo, Egypt

**Keywords:** Sinus-lifting, Osseodensification, Oblique sinus floor, Densah lift

## Abstract

**Purpose:**

To evaluate the effectiveness of using Densah burs for lifting the maxillary sinus membrane in cases with an oblique sinus floor with a residual bone height of 4–7 mm.

**Methods:**

The study was conducted on 16 patients, comprising 9 males and 7 females, aged 25–60 years, split into two groups of 8 each: group I with a residual bone height of 4–5.5 mm below the sinus floor and group II with a residual bone height of 5.5–7 mm. Exclusion criteria included smokers, presence of systemic or metabolic conditions that contraindicate implant placement and a local sinus pathology. The study involved the use of Densah burs, using the osseodensification concept to elevate the sinus floor, along with simultaneous dental implant placement. The integrity of the sinus membrane was verified via clinical examination and a confirmatory cone beam computed tomography scan.

**Results:**

The study revealed that out of the 16 cases, one case had a sinus membrane perforation, confirmed clinically at the time of the operation. The study achieved a mean lift of 4.42 mm and a mean final seating torque of 35.5 N/cm. At the 1-year follow-up, all cases showed clinical success, with no signs of sinus pathology or complications.

**Conclusions:**

In cases with oblique sinus floors and a residual bone height of 4–7 mm in moderately atrophic posterior maxilla, the osseodensification concept proved to be a safe and effective method for performing sinus lift procedures with simultaneous implantation.

**Graphical Abstract:**

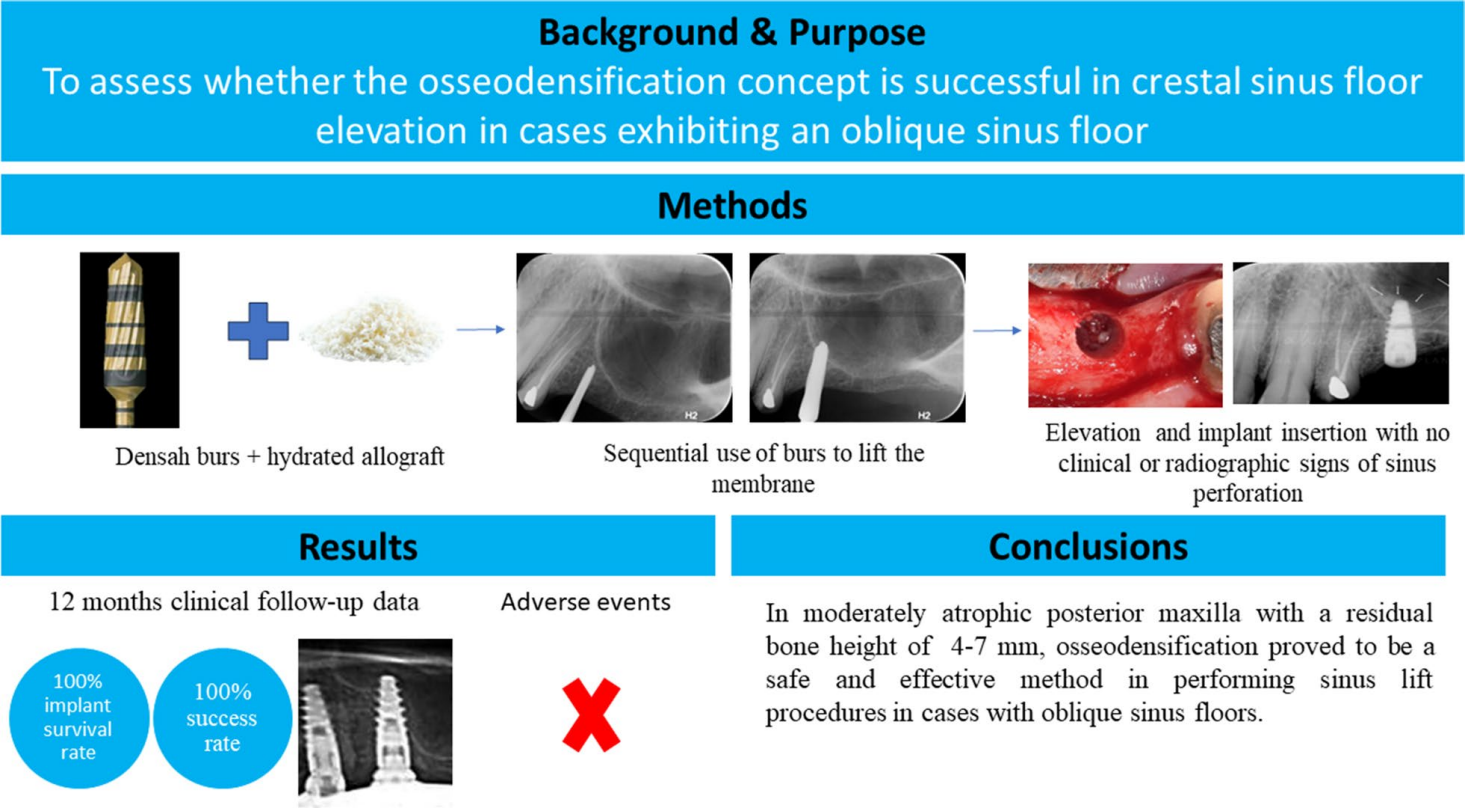

## Introduction

Sinus floor pneumatization following tooth extraction in the posterior maxilla commonly complicates the placement of dental implants. When feasible, short implants less than 8 mm in length have been shown to be successful in managing these cases [1]. However, in the majority of cases, sinus lifting is required for placement of dental implants of a standard length or in combination with short implants [2, 3]. Common practice has been to utilize a lateral window approach with or without simultaneous implant placement when the residual bone height (RBH) is 5 mm or less [4]. However, when the RBH is more than 5 mm, a crestal approach can be utilized [5, 6]. The crestal approach prevents most of the drawbacks associated with the lateral window approach, namely, the increased morbidity, risk of membrane perforation and the extended healing period required for the ossification of the used biomaterials [7, 8].

Crestal sinus floor elevation is a minimally invasive procedure with a lower complication rate compared to the lateral approach. Osteotome-mediated sinus floor elevation (OMSFE) was first described by Summers in the early nineties [9]. The technique witnessed numerous modifications afterwards, with and without the use of osteotomes and/or bone grafts [10–12].

Sinus membrane perforation is the most common complication associated with sinus floor elevation. A factor that complicates OMSFE further is that sinus pneumatization does not occur in a straight plane; in many cases, it leaves the sinus floor slanted, creating an oblique sinus floor. The floor at such regions makes an angle equal to or greater than 45 degrees with the rest of the floor. In such situations, the OMSFE was found to have a rather high rate of membrane perforation [13, 14].

The osseodensification (OD) technique uses a special set of burs that work by a non-excavating drilling process. Working in a counterclockwise direction, the burs are believed to cause low plastic deformation of trabecular bone [15]. This results in an accumulation of bone apically and laterally, which was found to be very successful in crestal sinus floor elevation with minimal risk of membrane perforation [16, 17].

The primary outcome of this current study aimed to assess the efficacy of elevating the maxillary sinus floor using Densah burs and the OD technique in atrophic posterior maxilla cases with a RBH of 4–7 mm and an oblique sinus floor. The secondary outcome was to assess the implant’s primary stability at the time of insertion. The null hypothesis stated that the use of Densah burs would not result in an increased membrane perforation rate when used for crestal sinus floor elevation in cases exhibiting an oblique sinus floor.

## Materials and methods

A total of 16 patients (9 males and 7 females with an age range of 25–60 years) meeting the eligibility criteria for crestal sinus floor elevation were included in this study. Inclusion criteria were as follows: RBH below the sinus floor of at least 4 mm and not more than 7 mm, with evidence of a sloping sinus floor on the panoramic view of the cone beam computed tomography scan (CBCT). Patients chosen were non-smokers and healthy without any systemic or metabolic conditions that may contraindicate dental implant placement. Patients should have no sinus pathology detected radiographically. The patients were further divided evenly into two groups: Group I: RBH 4–5.5 mm and Group II: RBH 5.5–7 mm. The rationale behind this patient grouping was to define the minimal RBH in cases with an oblique sinus floor at which the sinus membrane could be safely elevated without risk of perforation. A secondary outcome was to determine whether there would be a significant difference in implant primary stability between both groups.

CBCT (Soredex, Cranex, 3DX, Finland) was performed for every patient prior to the surgery to determine the bone height and width at the proposed implant site. For all cases, a dental implant of at least 8 mm in length and 4.3 mm in diameter was planned to be placed. The CBCT was also used to assess the bone density and the thickness of the Schneiderian membrane.

**Fig. 1:**
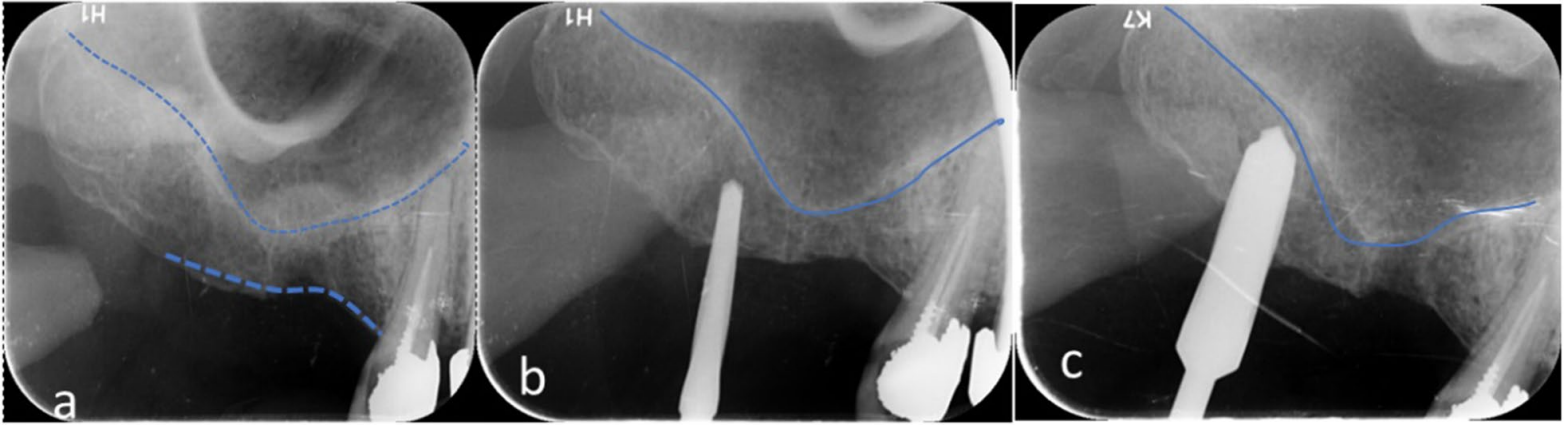
**a**–**c** Showing sloping sinus floor and sequence of drilling using the Densah burs

For all patients Amoxicillin/Clavulanate 1 g (Augmentin, GlaxoSmithKline, England) oral tablet was given 1 h before the surgery as an antimicrobial prophylaxis. In case of allergy, Clindamycin 600 mg (Dalacin C, Pfizer, USA) was prescribed. A mid-crestal incision along with mesial and/or distal sulcular incisions around the neighboring teeth was made, and a full thickness flap was raised at the area of interest. In each case, the implant osteotomy was initiated using a 1-mm pilot drill rotating in a clockwise direction at a speed of 900 RMP. The drill was initially advanced up to 1 mm below the lowest point of the oblique sinus floor as measured on the CBCT. A digital periapical radiograph (Digora, Sordex, Finland) was then taken immediately using a paralleling technique to ensure the drill position was correct. Once the position was verified, Densah burs (Versah LLC, USA) were used in the recommended sequence following the protocol specified in the densification guide recommended by the manufacturer for the implant system to be utilized. For a 4.3 mm implant, the Densah bur 2.3 mm was used in a counterclockwise motion with a drill speed of 900 RMP. When resistance was met due to increased bone density, the speed was increased gradually to no more than 1500 RPM. The bur was advanced in the osteotomy up to 0.5–1 mm below the oblique sinus floor following the path created by the pilot drill (Fig. 1)**.** This was followed by the 3.3 bur, which was then ready to breach the sinus floor. Following the haptic feedback that the sinus floor has been reached (bur being pushed out of the osteotomy as resistance is met), the bur was advanced in 1 mm increments, with care not to advance it more than 3 mm beyond the sinus floor to avoid tearing the sinus membrane. With this approach, autogenous bone chips from the osteotomy get packed around at the tip of the bur, facilitating the gentle separation and lifting of the membrane.

**Fig. 2:**
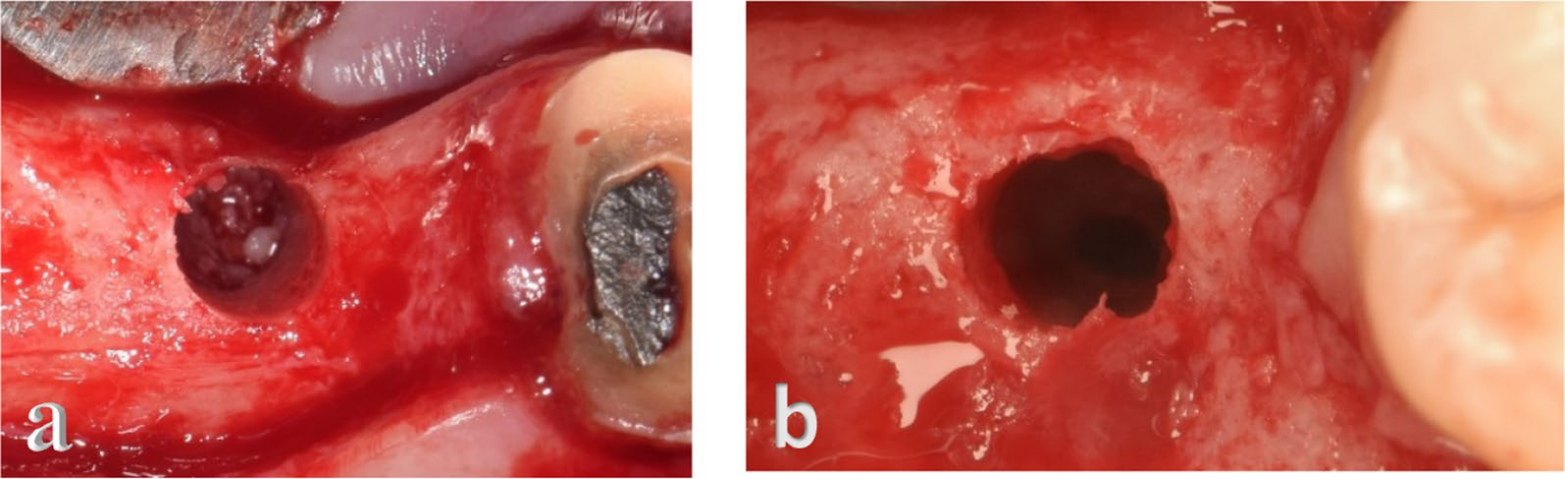
Sinus membrane integrity. **a** Intact **b** Perforated

Membrane integrity was verified visually under magnification using 6X dental loupes. An intact membrane expressed a greyish shadow with no discontinuity (Fig. 2a). A dark spot/hole was indicative of a perforation, which could range from a small tear to a complete perforation taking the whole circumference of the implant osteotomy (Fig. 2b). Saline irrigation was also passed several times through the osteotomy. The patient was asked if s/he felt fluid passing through the nasal cavity. If the patient started coughing or had a sensation of fluid in their nose, this was indicative of membrane perforation.

**Fig. 3:**
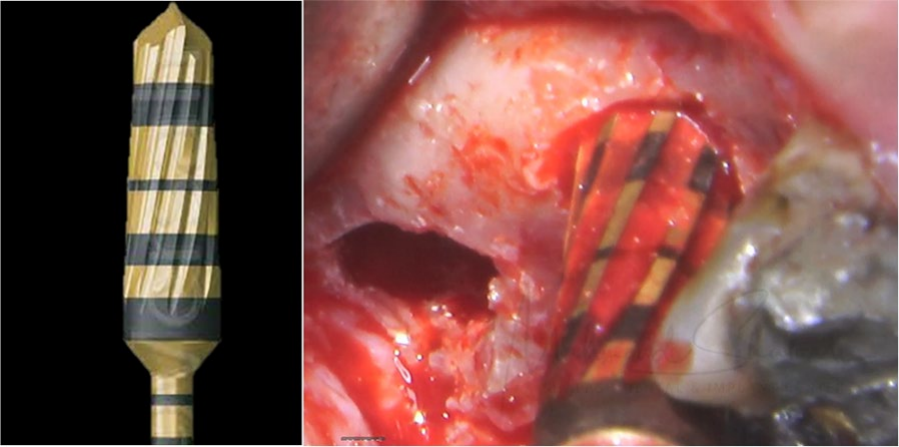
Showing Densah bur design

To complete the lift, 0.5 cc of hydrated allograft (Maxgraft, Botiss biomaterials GmbH, Germany) was applied in increments. The same 3.3 mm bur was used at a speed of 150 RPM without irrigation in a counterclockwise direction to propel the graft and further lift the membrane (Fig. 3). At all times, the bur was not advanced to more than 3 mm beyond the sinus floor.

**Fig. 4:**
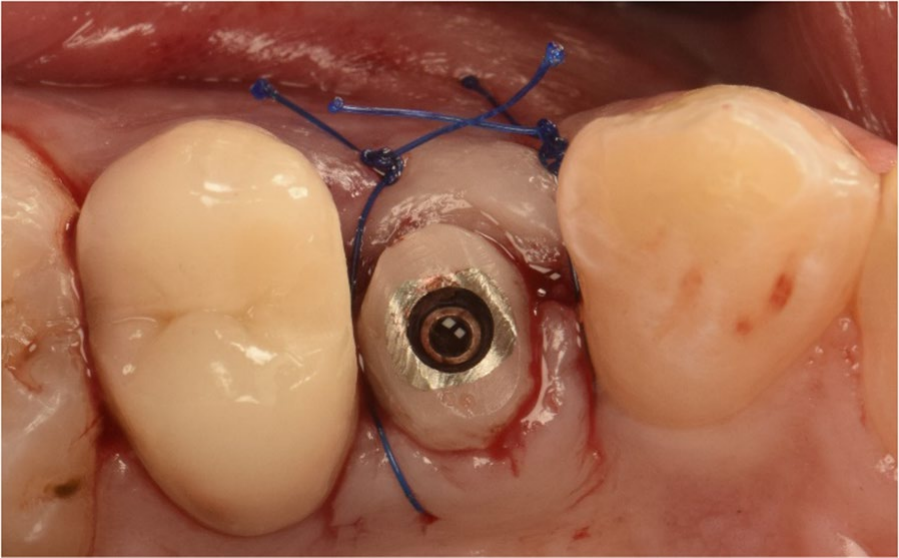
Custom healing abutment placed for a one-stage surge

In case a small tear in the sinus membrane was detected, a collagen sponge (Collacone, Botiss biomaterials GmbH, Germany) was inserted into the osteotomy. No graft material was added in this case. Following confirmed successful elevation, a 4.3-mm tapered internal hex implant, 8–11.5 mm in length was inserted. (JD Evolution Plus, JDentalCare, Italy). Implant primary stability was measured by inserting the implant using a motor-driven approach at a speed of 25 RPM (Implantmed, W&H, Austria). The motor placement facilitated the assessment of the final seating torque using the torque curve built-in function. A custom healing abutment fabricated chairside was attached in all cases for a one-stage surgery to shape the emergence profile and simplify the prosthetic phase of the treatment (Fig. 4). Flaps were approximated using 4–0 polypropylene sutures (Ethicon, Johnson & Johnson, USA).

**Fig. 5:**
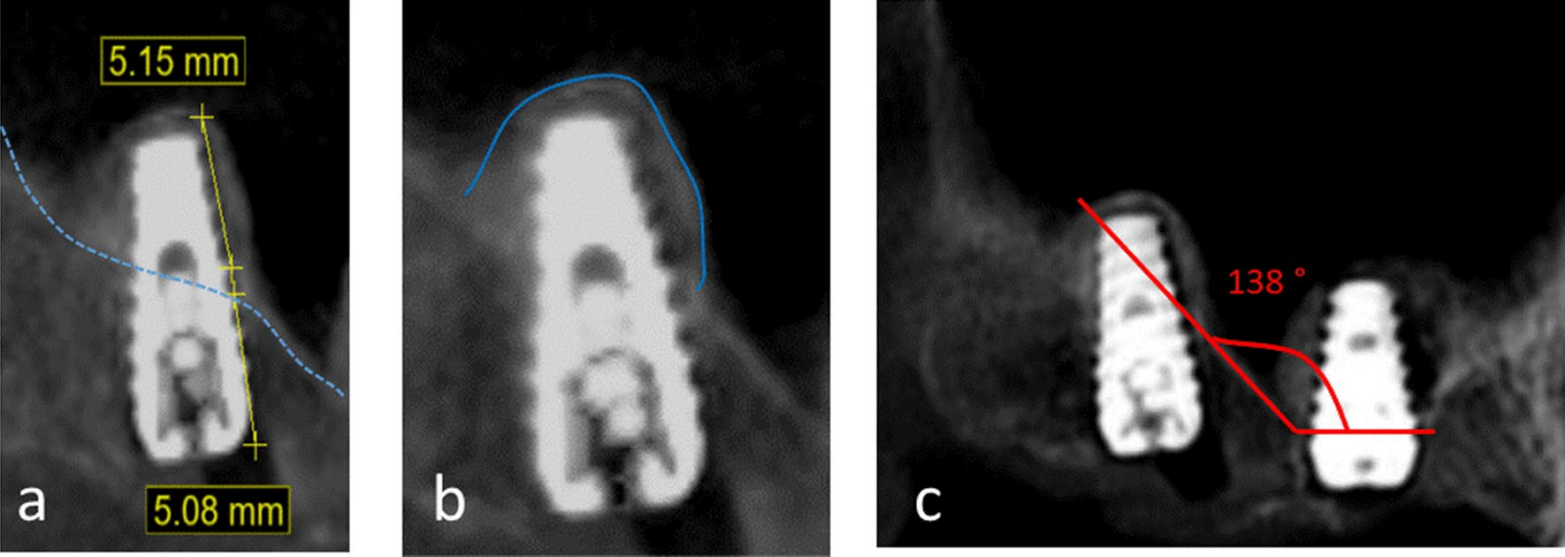
**a** Measuring the amount of bone gain achieved following the Densah lift. **b** Graft containment around the implant apex. **c** Angle measurement confirming a sloping sinus floor

Finally, an immediate post-operative CBCT was taken for each patient. The amount of elevation was measured by drawing a tangential line against the implant surface, just above the sinus floor, a measurement was taken up to the point where the graft material was seen covering the top of the implant apex (Fig. 5).

Standard post-operative instructions were given to all patients. Oral rinsing with chlorhexidine digluconate mouthwash 0.2% (Orovex, Macro Pharmaceuticals, Egypt) was prescribed for 10 days. An appointment for suture removal and follow-up was scheduled one week after the procedure. The final prosthesis was inserted 4 months following surgery.

### Statistical analysis

The mean and standard deviation values were calculated for each group in each test. Data were explored for normality using Kolmogorov–Smirnov and Shapiro–Wilk tests. Satisfaction data showed parametric (normal) distribution. Independent sample *t* test was used to compare between two groups in non-related samples. Pearson test was used to examine correlation between different parameters. The significance level was set at *p* ≤ 0.05. Statistical analysis was performed with IBM^®^ SPSS^®^ Statistics Version 20 for Windows.

## Results

A total of 16 patients (9 males and 7 females) with an age range of 25–60 years and a mean age of 42.5 years were treated in this study. Membrane perforation, confirmed clinically, occurred in one patient in group I. However, this perforation did not complicate or prevent implant insertion and was found to be statistically insignificant relevant to the Densah lift technique performed in all cases included in the study. In all the remaining cases, no sinus perforation was detected clinically or radiographically. The mean final seating torque and membrane elevation for group I & II are shown in Table 1. The amount of elevation was found to be significant with the highest mean value found in Group II and the lowest mean value found in Group I (*p* = 0.001 and *p* = 0.002 respectively). A 1-year follow-up following placement of the definitive restoration showed high patient satisfaction and no signs of implant- or sinus-related complications.

**Table 1:** The mean and standard deviation (SD) values of different parameters of different groups

Variables	Group I		Group II		*p* value
	Mean	SD	Mean	SD	
Final seating torque	33.71	1.50	37.38	1.92	0.001*
Elevation achieved	3.90	0.50	4.94	0.56	0.002*

*Significant (*p* < 0.05)

## Discussion

Crestal sinus floor elevation is a minimally invasive approach for placing implants in the posterior maxilla with reduced bone height with a low potential for complications [10, 18]. Many tools have been introduced to simplify the procedure and ensure successful lifting of the membrane with minimal trauma to the patient [19]. Membrane perforation remains to be the most commonly reported complication following crestal sinus floor elevation, with a reported perforation rate of 0–40% [20, 21].

A common challenge with the crestal approach is faced in sites exhibiting an oblique sinus floor. In such anatomic conditions, a crestal approach using an osteotome is associated with a higher risk of perforation. The reason for this lies in the direction of penetration of the osteotome, which enters the sinus cavity first at the lower level of an oblique sinus floor, with bone resistance still present on the higher level. By the time the osteotome reaches the higher bone level, it is already a few millimeters within the sinus, increasing the risk of perforation [13].

In our study, 16 cases with an oblique sinus floor with a RBH ranging from 4 to 7 mm were successfully treated with a closed sinus lift approach using Densah burs. The use of these burs in crestal sinus lift has become a well-established minimally invasive technique with reduced complication rate, compared to other sinus lifting techniques [16, 22, 23]. These burs with their unique flute design and counterclockwise rotation allow for autogenous bone compaction laterally along the walls of the osteotomy as well as apically toward the sinus floor. This compaction together with modulated pressure during drilling and the constant in-and-out pumping motion ensures that this well-hydrated autogenous bone creates a hydraulic pressure to gently lift and free the Schneiderian membrane. Further addition of a hydrated allograft material along with the propelling action of the burs gently pushes the graft against the membrane facilitating its lift [16, 17, 22, 24, 25]. We believe that these unique features in the bur design and the concept of OD have simplified the approach to a common problem in crestal sinus floor elevation, namely the oblique sinus floor.

One case in group I showed clinical signs of perforation. This could be attributed in part to the reduced RBH in this group (4–5.5 mm), which has been shown to be associated with increased chances of membrane perforation. Boyacıgil et al. showed that there was a higher rate of membrane perforation when the RBH was less than 5 mm [26]. Similar findings were also reported by Tükel & Tatli [27]. In our study, the case with the perforation had a RBH of 4.08 mm, which, in addition to the presence of an oblique sinus, could explain the possible reason for the perforation that occurred. Following the protocol proposed by Toffler M**,** the implants were placed without bone grafting in this case following the insertion of a collagen sponge into the perforation [28]. It could be concluded and based on the patient grouping that a safe range of elevation when an oblique sinus floor is encountered should be above 4 mm.

Numerous publications have shown that implants penetrating the sinus can have a favorable outcome with the membrane healing and becoming thickened around the implants in most cases [29, 30]. In a canine model, several studies assessing sinus health after the placement of dental implants that penetrated the sinus at variable distances ranging from 2 to 8 mm also showed absence of sinus complications and did not prevent successful implant osseointegration [23, 31, 32]. In our case with the perforation, the implant healed uneventfully with no clinical or radiographic signs of sinus complications at the 1-year follow-up session.

In our study, a mean elevation of 4.42 mm was achieved in both groups, allowing the placement of standard-length implants. This conforms to the results of other studies showing an average safe limit for the Schneiderian membrane elevation to be within 4–5 mm with minimal risk of perforation [33–35]. Despite our results showing a statistically significant difference in the elevation achieved in group II compared to group I, this difference was clinically insignificant since the mean elevation in group I also facilitated the placement of standard-length implants.

A secondary outcome in our study was the measurement of the implant’s primary stability at the time of implant insertion. OD is based on the preservation and collection of autogenous bone within the implant site via non-subtractive drilling and the compaction of cancellous bone, which has strong viscoelastic and plastic deformation properties [36]. Unlike traditional osteotomies, OD creates the osteotomy while preserving vital bone tissue and increasing the amount and density of peri-implant bone, in addition to increasing the bone-to-implant contact and percentage of bone volume around it, thereby improving implant stability [22, 37]. In our study, a mean final seating torque of 35.5 N/cm was achieved for all implants. Group II results showed a statistically significant difference in terms of primary stability compared to group I; these results, however, were clinically insignificant and could be attributed to the slightly higher RBH, which theoretically may have led to an increased bone-to-implant contact and hence, a higher implant primary stability.

For both groups, a connection of a custom healing abutment for a one-stage surgical approach was done, and an excellent final seating torque was established. This was very convenient to the patient as it meant less surgical intervention and less overall treatment time. Schiegnitz et al. [38] showed that in general, the oral health-related quality of life (OHRQoL) is markedly improved following maxillary sinus augmentation surgery.

One limitation to our study is the lack of a control group, an OMSFE used in a similar manner to elevate the Schneiderian membrane in an oblique sinus floor. Another variable that could have been tested is whether there is significant difference in the results between a grafted and a graftless elevation in cases with oblique maxillary sinus floors.

## Conclusion

To our knowledge, this is the first study assessing the clinical outcomes of using Densah burs in an oblique sinus floor. With a limited sample size, our study has shown that in posterior sites with moderate RBH and a sloping sinus floor, Densah lift using the OD concept is a successful technique without an increased risk of Schneiderian membrane perforation. A 1-year follow-up post-loading showed no complications with 100% implant survival rate. Further studies with larger study samples and study groups are recommended.

## Data Availability

The data that support the findings of this study are available from the corresponding author upon reasonable request.
